# A Model of Evolution of Development Based on Germline Penetration of New “No-Junk” DNA

**DOI:** 10.3390/genes3030492

**Published:** 2012-08-02

**Authors:** Alessandro Fontana, Borys Wróbel

**Affiliations:** 1 Faculty of Electronics, Telecommunications and Informatics, Technical University of Gdańsk, Gabriela Narutowicza 11/12, PL80233, Gdańsk, Poland; 2 Evolving Systems Laboratory, Faculty of Biology, Adam Mickiewicz University, Umultowska 89, PL61614 Poznań, Poland; 3 Systems Modelling Laboratory, Institute of Oceanology, Polish Academy of Sciences, Powstańców Warszawy 55, PL81712, Sopot, Poland; 4 Institute of Neuroinformatics, University & ETH Zurich, Winterthurerstrasse 190, CH8057 Zurich, Switzerland

**Keywords:** transposable elements, embryonic development, evo-devo

## Abstract

There is a mounting body of evidence that somatic transposition may be involved in normal development of multicellular organisms and in pathology, especially cancer. Epigenetic Tracking (ET) is an abstract model of multicellular development, able to generate complex 3-dimensional structures. Its aim is not to model the development of a particular organism nor to merely summarise mainstream knowledge on genetic regulation of development. Rather, the goal of ET is to provide a theoretical framework to test new postulated genetic mechanisms, not fully established yet in mainstream biology. The first proposal is that development is orchestrated through a subset of cells which we call driver cells. In these cells, the cellular state determines a specific pattern of gene activation which leads to the occurrence of developmental events. The second proposal is that evolution of development is affected by somatic transposition events. We postulate that when the genome of a driver cell does not specify what developmental event should be undertaken when the cell is in a particular cellular state, somatic transposition events can reshape the genome, build new regulatory regions, and lead to a new pattern of gene activation in the cell. Our third hypothesis, not supported yet by direct evidence, but consistent with some experimental observations, is that these new “no-junk” sequences—regulatory regions created by transposable elements at new positions in the genome—can exit the cell and enter the germline, to be incorporated in the genome of the progeny. We call this mechanism germline penetration. This process allows heritable incorporation of novel developmental events in the developmental trajectory. In this paper we will present the model and link these three postulated mechanisms to biological observations.

## 1. Introduction

A large part of the genomes of multicellular organisms corresponds to transposable elements (TEs). For example, long interspersed element 1 (LINE1 or L1) comprise about 17% of the human genome (with more than half a million copies), and in addition contribute indirectly (through non-autonomous partners of L1, Alu and SVA) to another 10% [1]. About 80–100 L1 elements are retrotransposition-competent in humans [2]. These elements have been active in the mammalian lineage for at least 100 million years [1] and continue to be active in most mammals tested thus far (with possible exception of some rodents and bats; [3,4]).

Recent experimental evidence suggests that somatic L1 transposition may be a part of the developmental programme, and may occur at very early developmental stages; indeed it appears to be necessary for early embryogenesis [5]. Other data suggest that somatic L1 retrotransposition happens also later, resulting in an increased number of insertions in specific tissues (in particular, in some brain regions, in the order of thousands insertions; [6,7]).

Such somatic transposition is very likely tightly regulated, and otherwise TEs are silenced in the cells by various mechanisms, including DNA methylation and packaging into inactive chromatin, but these mechanisms may break down in cancer cells [8]. However, transposition can also occur in the germline, creating a heritable variation in the number of TE copies in the genome. In the case of L1 and its non-autonomous partners such insertions happen perhaps as frequently as every 20 births [9].

The current view is that (i) somatic transposition does not affect the germline, unless it occurs very early during development; and (ii) the insertion events affecting the germline are essentially random. In this paper we present a conceptual model which breaks with these two assumptions. We provide a description of plausible biological mechanisms which are consistent with our model and present a software platform in which similar mechanisms have been implemented, resulting in a system for modelling evolution of embryogenesis.

This system is called Epigenetic Tracking (ET, [10,11,12,13,14]) and belongs to the field of Artificial Embryology. As the name implies, the interest of Artificial Embryology is in building artificial systems inspired by the mechanisms of biological development. This can be seen as a way to inform biology by providing the summary of existing knowledge, understanding-by-building, or an opportunity for hypothesis testing, but also an inspiration for the experimental search of new biological mechanisms. More engineer-minded Artificial Embryology aims at bio-inspired artificial systems which would be self-constructing, self-repairing, and robust to damage.

**Figure 1:**
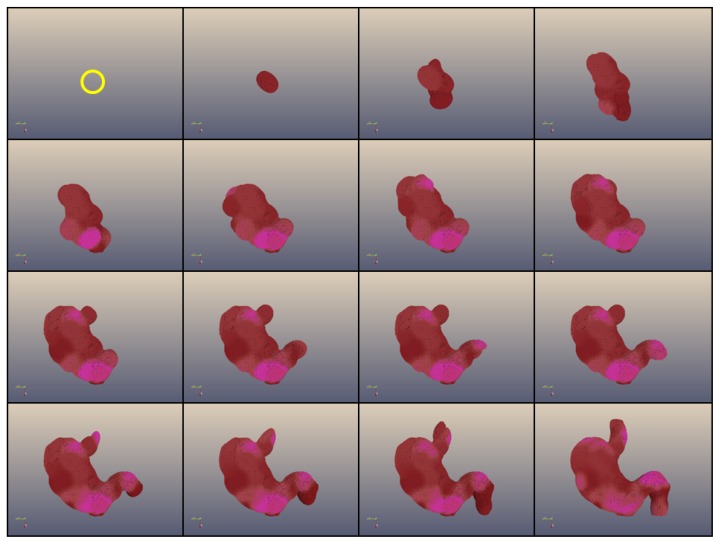
Development of a 3D coloured shape (a “human stomach”) composed of 1,000,000 cells obtained with Epigenetic Tracking. The frames represent the development of an individual obtained in a simulated evolutionary process which started from a population of individuals with random genomes. The population in each generation *n* + 1 was created by choosing (with mutation and recombination) individuals from the population at generation *n* based on their fitness, corresponding to the proximity of the developed body to the shape representing a human stomach. Development starts from a single cell (in a yellow circle in the first frame) and proceeds through 15 developmental stages. The figure shows the development of the most fit individual after 80,000 generations of simulated evolution.

There has been a number of AE platforms which are built on the concept of artificial gene regulatory networks encoded in linear genomes (e.g., [15,16]). ET is more abstract, but nonetheless there is a plausible correspondence between the elements of the system and such biological entities as coding genes or regulatory regions. ET allows the “devo-evolution” of very large artificial multicellular “bodies” with complexity (in terms of the number of cells and the level of detail) unprecedented in the field of Artificial Embryology (Figure 1). In the next section we will present the elements of our conceptual model of development while illustrating how various concepts could relate to biological entities and the elements of our artificial system. We will then discuss the possible implications of our model.

## 2. Epigenetic Tracking as a Model of Development

The key feature of ET is the existence of a small subset of cells (which we call *driver* cells) which orchestrate developmental events at a particular time during development. Time is measured in ET in developmental stages. We use an abstraction for measuring developmental time, a variable called global clock (GC), to count the stages. This is a convenience of our software implementation, other ways of timing developmental events would work equally well.

**Figure 2:**
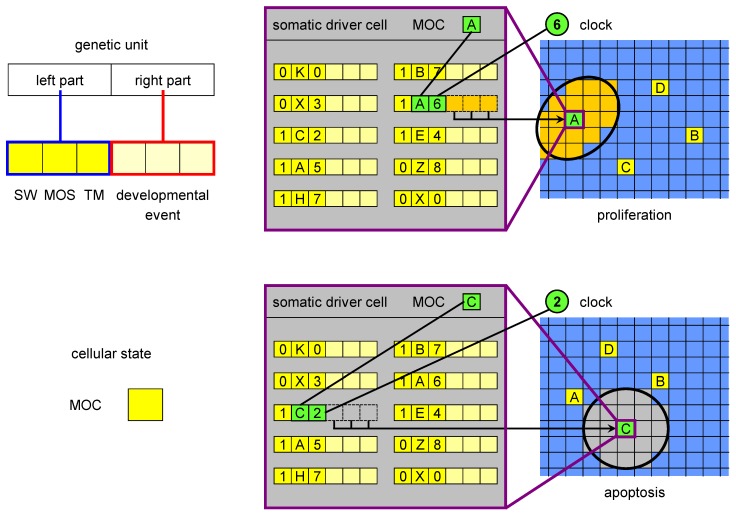
The MOC, genetic units and developmental events in ET. The MOC (mobile code) is an abstraction of the cellular state, consisting of all proteins and RNAs acting in gene regulation (as trans factors). The left part of a genetic unit is composed of three elements: SW (switch), MOS (mobile sequence) and TM (timer). The MOS is an abstraction of all regulatory loci (cis regulators) regulatory factors can bind to. The right part encodes a developmental event, which is triggered when i) SW is in the active state, ii) there is a match between the MOS of the unit and the MOC of the driver cell and iii) there is a match between the clock and the timer value. There are two types of developmental events: proliferation and apoptosis. The figure shows an example of a proliferation event triggered by the driver cell with MOC labelled with A at developmental stage 6 (when the clock value is 6) and of an apoptosis initiated by MOC C at stage 2.

In ET (Figure 2), driver cells cause either a large-scale apoptosis (death of a large number of cells in the vicinity of a driver cell, called *change volume*), or a proliferation event, which corresponds to several rounds of cell divisions and results in the formation of a large number of cells in the change volume. Some of these cells are stem cells which later produce terminally differentiated cells, and some are drivers having a new state, different with respect to the original driver that triggered the developmental event. 

Many analogies exist between the concept of a driver cell and that of embryonic “organisers”, such as the Spemann’s organiser, an area of the embryo able to induce embryonic primordia upon transplantation into a different location [17,18]. Similarly, if a driver cell destined to give rise to a certain part of the structure were transplanted to a different position of the growing structure, such part would grow in this ectopic position. In ET a single driver cell, when triggered to proliferate, induces the creation of other driver cells; some of these will become the centres of other proliferation or apoptosis events, from which other driver cells are created, until the whole structure is generated. This appears to be coherent with the so-called “head/trunk/tail organiser model” [17], which foresees the presence of multiple organisers. In ET the organisers (the driver cells) are many. They are hierarchically structured and are continuously created during development.

**Figure 3:**
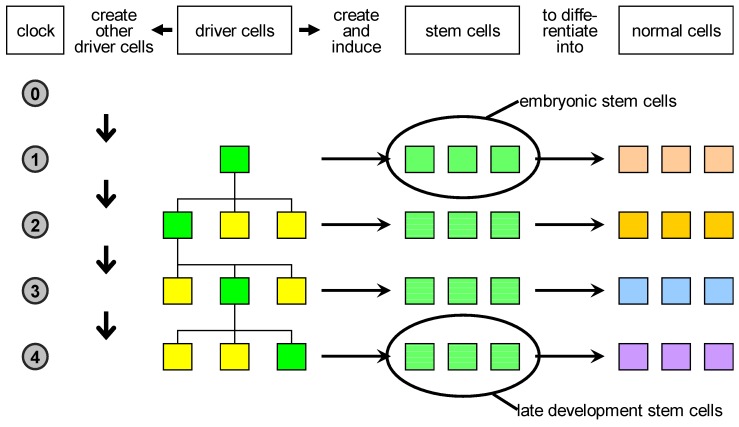
Proposed classification of natural cells. Driver cells marked in green become active during development and give rise to proliferation or apoptosis events. In case of proliferation, they produce a local pool of stem cells, specific for the body part under construction, that are later induced to differentiate into the specialised cell types needed. In parallel, other driver cells are generated and homogeneously distributed in the part just created: some of those are destined to become centres of other developmental acts in the course of development.

Based on these considerations, we propose a reclassification of biological cells (Figure 3):

1. driver cells. These are the precursors of stem cells and correspond to biological organisers. They can create and/or induce stem cells to commit to specific fates;
2. stem cells. These are cells characterised by a high degree of plasticity and are susceptible to be induced by driver cells to take different cellular fates;
3. normal cells. These are cells with no plasticity, *i.e*., terminally differentiated cells. Actually, stem cells and normal cells can be imagined as lying on a continuum of cells characterised by decreasing plasticity.

The key novelty here is the proposal of the existence of the driver cells, which represent the scaffold of the developing organism. 

We hypothesise that if a driver cell enters a particular state at a particular time (in our implementation of ET, this is the combination of the clock (GC), and a variable called mobile code (MOC), Figure 2), it expresses a set of genes. We understand the state of a biological cell as a composite of epigenetic marks, the chromatin state, the concentrations of regulatory proteins and RNAs (and their posttranscriptional/posttranslational modifications), and of other regulatory substances, *etc*. When the driver cell expresses the genes corresponding to this regulatory millieu, a particular developmental event is orchestrated. As an abstraction of the process in which this set of genes is expressed, we use the match between the MOC and another variable called mobile sequence (MOS, which belongs to the left part of genetic units in ET, Figure 2), and between GC and TM (timer, also a field of the left part). The genes are not expressed if the SW field (for switch) has a value corresponding to the inactive state. In biological terms such state can be understood as pseudogenisation of an essential regulatory factor. The developmental event is specified by the right part of the genetic unit.

As was expressed above, there may be various molecular mechanisms for the election of an event based on the state of the driver and the developmental time. We will use a shorthand of a match between the “biological MOC” and “biological MOS” from now on to refer to what we consider to be an important subset of these mechanisms. The nomenclature we use (“mobile code” and “mobile sequence”) stems from the hypothesis that many regulatory regions in the genomes of multicellular organisms may derive from recent or ancient transposition events [19]. Our hypothesis is that such new regulatory regions that allow the driver cells to orchestrate developmental events are constantly generated through the activity of mobile DNA, and are a part of the pool of sequences which seem to be “junk DNA”, but play an important functional role in development and evolution. In order to emphasise the match between such “no-junk mobile sequences” and the state of the cell—a code of regulatory proteins, RNAs, *etc*.—we use the terms “mobile sequences” (MOS) and “mobile code” (MOC). A lack of match between biological MOC and MOS may correspond to a lack of some regulatory elements in the vicinity of genes which are necessary for an event to happen.

## 3. Germline Penetration: The Link between Development and Evolution

Most driver cells produced during development do not orchestrate any events (are inactive). In ET this may correspond to a driver cell which has a MOC that corresponds to a MOS in a right part of some genetic unit but the TM in this right part has a value higher than the maximum number of developmental stages, or the driver is produced at a stage which is “later” than any of these TMs, or all the genetic units with a matching MOS have the field SW set to “inactive”. A simple germline mutation may change the situation.

It is also possible that some driver cells are inactive because there is no MOS in the genome which would match their MOC. In biological terms it may mean that in this particular state of the driver, all the regulatory factors (proteins, RNA) present in the cell do not result in the activation of all the genes which are necessary to allow for an event to happen. In ET MOCs and MOSs are encoded as long sequences of numbers. Therefore, the probability that a suitable MOS emerges in the genome simply through mutations and recombinations is very low.

**Figure 4:**
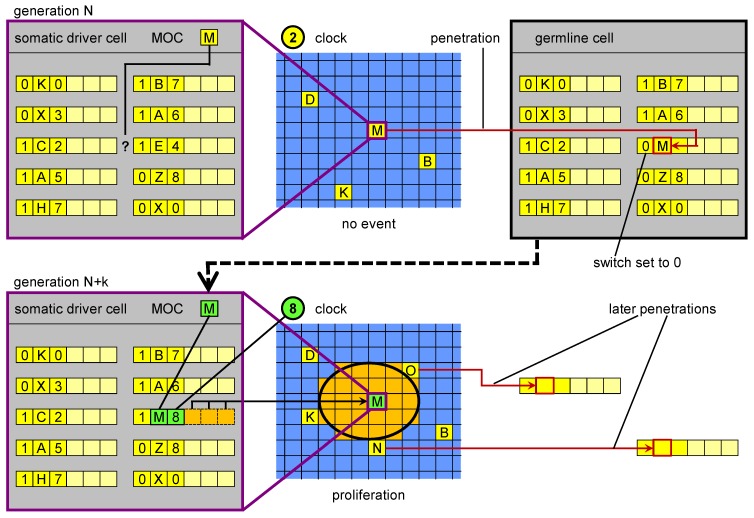
Germline penetration of new regulatory sequences. In the upper part, in a driver cell the genome is not able to “respond” to the current cellular state M with a corresponding event. Transposition events create a new MOS that is able to respond. The new MOS leaves the driver cell and reaches the germline, where it is incorporated in the genome. In a subsequent generation, when a cell reaches cellular state M, its genome (inherited from the penetrated germline) can respond with a specific genetic unit. The result is a proliferation event.

The procedure that allows to overcome this hurdle is called *germline penetration* in our conceptual model. It consists in the introduction into the genome of new genetic units which have a MOS corresponding to a MOC of an inactive driver. The SW field is initially set to “inactive” (Figure 4). Otherwise, the penetrated genetic units would all become active with a non-optimised right part, causing a major disruption in the individual’s development: their activation is obtained through a subsequent, “standard” mutation. To sum up, the mechanism of germline penetration is needed in ET because of the way genetic units are encoded in the genome. Our experiments *in silico* (not shown) demonstrate that when germline penetration is disabled, the evolutionary process practically grinds to a halt.

In biology the lack of a matching MOS may correspond to a situation in which a lack of only one regulatory region may make a set of active genes incomplete for the activation of a specific event. If this is so, one random mutational event could change the situation. However, the observation of somatic transposition resulting in the potential formation of new regulatory regions offers another possibility. Our postulate is that an equivalent of germline penetration in ET (a process in which new MOSs are generated in somatic driver cells and then migrated to the germline) exists in biological systems. Moreover, we propose that this process allows for higher evolvability. Physical penetration may occur in biology perhaps thanks to endogenous viruses or circulating nucleic acids.

Our proposed mechanism for this process is two-part. First, new regulatory regions are created through somatic transposition, in effect producing missing parts of biological MOSs in driver cells. If such a new MOS matches a MOC and is actually used for proliferation, the number of cells carrying the new regulatory region will expand. We hypothesise that as a consequence there will be higher chance that the new regulatory element physically penetrates to the germline. If the developmental event is apoptosis, it is possible that the DNA released from the cells enters the blood stream, which also increases the chances of penetration. We hypothesise that the fact that the postulated formation of new regulatory regions occurs thanks to transposition events makes them more mobilisable for germline penetration. This could involve a process similar to 3’ transduction of SVA and L1 elements (which affects about 10% of their insertion events, [9]).

We propose this mechanism inspired by the fact that germline penetration greatly increases evolvability in ET. However, the fact that this feature plays such an important role in a software simulation cannot be presented as evidence for the existence of its biological equivalent. It is possible that its importance is an artifact caused by the way in which MOS and MOC are represented: as digital numbers whose random match would be extremely unlikely. In our software when no match is found for a given MOC, a driver cell that has it stays developmentally dormant. We think (but without any actual evidence) that such dormant cells have parallels in biology. We propose, moreover, that in these cells the activity of mobile DNA is particularly pronounced, allowing for the generation of new MOS sequences. Our hypothesis is that this generation is helped by the repertoire of the regulatory factors in the cell, which helps in the creation of a biological MOS responsive to a particular biological MOC.

At this point we cannot present any experimental evidence which would support the view that in biology, as in our software simulation, random transposition events in the germline are insufficient to generate the regulatory sequences (a biological MOS) which would give relevant novel developmental events after novel cellular states (MOCs) are created in driver cells during development. The same goes for random somatic transposition/mutation events in the lineage of a driver (resulting in MOSes irrelevant for the MOC of a driver in the dormant state). In the latter case, the effect of penetration would be the same as the effect of transposition and mutation in the germline. In other words, it is possible that because the biological MOS-MOC matching is based on different principles than the way the matching was implemented in our software, finding a new match through random events is much more likely in biology. If this were so, than biological germline penetration would not be necessary per se. We do believe, however, that if such a mechanism existed, it would allow for increased evolvability, as in our software simulation. Moreover, there are a number of biological observations which are consistent with a hypothesis of biological germline penetration for which we advocate in this paper.

## 4. Biological Observations Consistent with Germline Penetration

An increased activity of TEs in the germline [20] is coherent with our hypothesis. Because only TEs which become fixated in the germline can be passed on to the next generation and have trans-generational effects, this increased activity may correspond to the insertion of new regulatory DNA generated during development. Again, direct evidence is lacking, but according to our hypothesis, it would be the final stage of germline penetration. As pointed out in [5], TE activity peaks in the germline and remains high in the early stages of embryogenesis, to quickly fade away when the embryo reaches the blastocyst stage. These observations are coherent with our model if we assume that sequences which penetrate to the germline are maintained as extrachromosomal structures and their insertion continues in the embryo.

**Figure 5:**
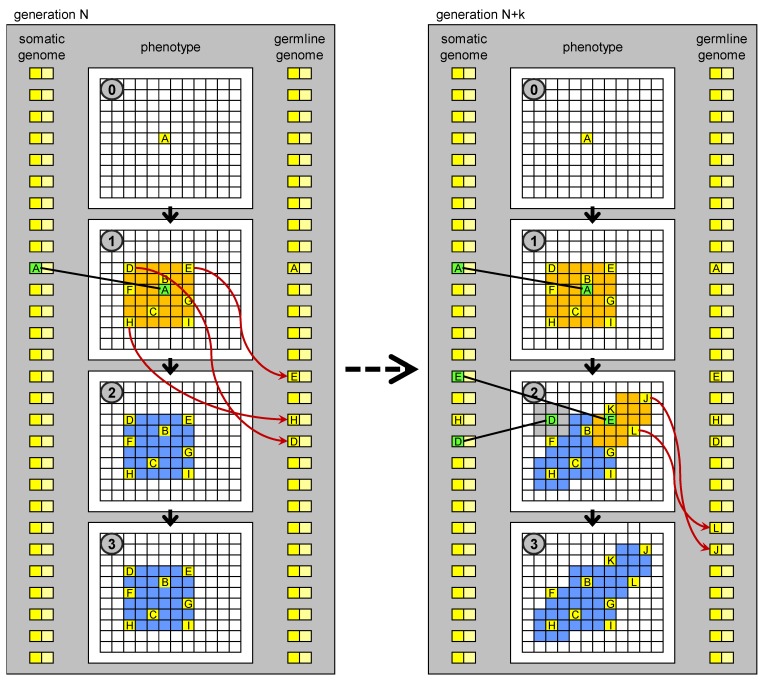
Evolution of development based on germline penetration of new “no-junk” DNA. On the left: development of an individual at generation *N*. Development stops at stage 1. New MOS sequences derived from transposition events (new “no-junk” DNA) matching particular states (MOC) of drivers cells, which orchestrate the development, are transferred to the germline genome. The germline genome is inherited by the individuals in the next generation. On the right: development of an individual at generation *N* + *k*. New genetic units, derived from the penetrated elements, are present in the genome, whose regulatory sequences match some of the MOC sequences generated in stage 1, so development can proceed further than at generation *N*. The new MOS sequences generated in stage 2 can again penetrate to the germline genome.

The term “germline penetration” draws inspiration from the hypothetical process in which the genetic sequences generated during development in driver cells are transferred to the germline, perhaps carried in the blood stream. Whenever a proliferation occurs as a result of the appearance of a new biological MOS in a (somatic) driver cell, a “wave” of new cells (driver, stem, and normal) appears, each with this new MOS. Germline penetration allows the spray of this wave to reach the germline genome, and subsequently the genome present in all the cells (somatic and germ) in the next generation (Figure 5). If such a process exists, it may have been a key innovation in the evolution of multicellular development in the history of life on Earth.

The moments of the penetration events during evolution would coincide with changes to the developmental trajectories. In principle, such changes could result in the appearance of new body parts, although most such drastic changes would be lethal to the embryo. It is thus expected that most of such changes correspond to enlargement or shrinkage of existing organs or their parts. Such alterations of development can, nonetheless, have profound consequences, resulting in the generation of new species, and the eventual growth of new branches of the “tree of life”. The postulate that the generation of new MOS is based on the proliferation of TEs in the genome is in agreement with the hypothesis that speciation is associated with the expansion of repeatable DNA in the genome [19,21].

One part of the actual mechanism in which new MOS penetrate to the genome could be circulating nucleic acids or endogenous viruses. Nucleic acid circulating in the blood range from 500 bp to more than 30 kb and are released from cells in complexes with proteins, proteolipids and/or in vesicles [22]. This cell-free DNA contains repetitive sequences, including LINE1 [23]. The concentration of DNA in the blood is increased in many cancer patients [22,23], and it was proposed that such DNA released from primary tumour cells could lead to metastases in distant organs through a transfection-like uptake [24].

Another part of the mechanism of germline penetration could be the process called Sperm-Mediated Gene Transfer (SMGT) [25]. SMGT is a process that allows the introduction of new genetic traits in animals by exploiting the ability of spermatozoa to take up exogenous nucleic acid molecules and deliver them to oocytes at fertilisation. The experimental evidence suggests that SMGT is a retrotransposon-mediated phenomenon.

The conjecture of the existence of a biological germline penetration is grounded on the relevance that this procedure has for the evolvability in our computational simulations using ET, where it is indispensable for the evolution of multicellular structures. This point deserves emphasis: in the case of the evolution of complex multicellular “bodies” in this system, random mutational process *is not sufficient*. When germline penetration is switched off, evolution halts in our system. The process of germline penetraton provides a more tight coupling between development and evolution than currently thought. If somatic genetic innovations are transferred to the germline genomes as we hypothesise, then not only “nothing in biology makes sense except in the light of evolution”, but “nothing in multicellular biology makes sense except in the light of devo-evolution”.

Additional biological evidence consistent with our hypothesis is the proposal for high TE activity in the brain during embryonic development, in comparison with other tissues and organs [26], so far interpreted as the outcome of *random* transposition events, possibly explaining different performance of genetically identical animals in the same behavioural task [27]. From an evolutionary viewpoint, this phenomenon is interpreted as a potential source of genetic variation produced during development which could help individuals to cope with rapidly changing environmental conditions.

According to our conceptual model, somatic transposition during development is the main source of evolutionary innovations, so it should happen in all the organs (or their primodia). But if it is more prevalent in the brain, it is interesting to ask why. One explanation is that it does indeed occur at a higher rate in the brain tissues, and as a consequence the brain and the behavioural traits evolve faster. This may be because of the higher pressure for such evolvability. It has been observed that cells in which transposition occurs may be a target of the immunological system, because the higher chance of genomic instability brings a higher risk of cancer. The trade off between evolvability and carcinogenesis may result in a different optimal allowed rate of transposition in the brain because it contains a large number of cells in which cell division is arrested. It can also be affected by the fact that the immunological response in the brain works differently than in the rest in the body. Alternatively or in addition, the brain-blood barrier may also mean that less of the newly generated MOS can reach from the brain to the germline, and the higher rate of the generation of such MOS is needed to compensate and maintain higher evolvability.

## 5. Conclusions

We have proposed a conceptual model of development, Epigenetic Tracking, in which somatic transposition generates developmental innovations which are then transferred to the germline. We have previously shown in software simulations that the implementation of this model allows for the evolution of developmental processes which can generate highly complex 3-dimensional structures starting from a single cell. In previous work ET was used to model a simplified version of key biological phenomena such as the role of pseudogenes in evolution, the phenomenon of ageing and the process of carcinogenesis. In this work, we draw from the importance of the process of germline penetration for the evolvability in ET in order to formulate a hypothesis of how new “no-junk” regulatory sequences resulting from biological somatic transposition could be transferred to the germline and thus become a source of heritable developmental innovations. Biological sciences, including Evolutionary Biology and Systems Biology, traditionally use a “bottom-up” approach which starts from genes and proteins and tries to bring understanding through building of complex models. Our work is informed by a “top-down” approach that proceeds from models of high-level mechanisms towards a proposal for biological, low-level molecular processes. We argue that both approaches have merits and the hope that the two paths can cross midway.
